# Non-Oncological Neuroradiological Manifestations in NF1 and Their Clinical Implications

**DOI:** 10.3390/cancers13081831

**Published:** 2021-04-12

**Authors:** Camilla Russo, Carmela Russo, Daniele Cascone, Federica Mazio, Claudia Santoro, Eugenio Maria Covelli, Giuseppe Cinalli

**Affiliations:** 1 Department of Electrical Engineering and Information Technology (DIETI), University of Naples “Federico II”, 80125 Naples, Italy; 2 Pediatric Neuroradiology Unit, Department of Pediatric Neurosciences, Santobono-Pausilipon Children’s Hospital, 80129 Naples, Italy; c.russo@santobonopausilipon.it (C.R.); d.cascone@santobonopausilipon.it (D.C.); f.mazio@santobonopausilipon.it (F.M.); e.covelli@santobonopausilipon.it (E.M.C.); 3 Neurofibromatosis Referral Center, Department of Woman, Child, General and Specialized Surgery, Università degli Studi della Campania “Luigi Vanvitelli”, 80138 Naples, Italy; claudia.santoro@unicampania.it; 4 Clinic of Child and Adolescent Neuropsychiatry, Department of Mental and Physical Health, and Preventive Medicine, Università degli Studi della Campania “Luigi Vanvitelli”, 80138 Naples, Italy; 5 Pediatric Neurosurgery Unit, Department of Pediatric Neurosciences, Santobono-Pausilipon Children’s Hospital, 80129 Naples, Italy; g.cinalli@santobonopausilipon.it

**Keywords:** phakomatosis, neurofibromatosis type 1, central nervous system, SPINE, computed tomography, magnetic resonance imaging

## Abstract

**Simple Summary:**

Central nervous system involvement (CNS) is a common finding in Neurofibromatosis type 1 (NF1). Beside tumor-related manifestations, NF1 is also characterized by a wide spectrum of CNS alterations with variable impacts on functioning and life quality. Here, we propose an overview of non-oncological neuroradiological findings in NF1, with an insight on pathophysiological and embryological clues for a better understanding of the development of these specific alterations.

**Abstract:**

Neurofibromatosis type 1 (NF1), the most frequent phakomatosis and one of the most common inherited tumor predisposition syndromes, is characterized by several manifestations that pervasively involve central and peripheral nervous system structures. The disorder is due to mutations in the NF1 gene, which encodes for the ubiquitous tumor suppressor protein neurofibromin; neurofibromin is highly expressed in neural crest derived tissues, where it plays a crucial role in regulating cell proliferation, differentiation, and structural organization. This review article aims to provide an overview on NF1 non-neoplastic manifestations of neuroradiological interest, involving both the central nervous system and spine. We also briefly review the most recent MRI functional findings in NF1.

## 1. Introduction

Neurofibromatosis type 1 (NF1), the most frequent phakomatosis and one of the most common inherited tumor predisposition syndromes, is a multi-organ autosomal dominant disease with an incidence ranging between 1:2000 and 1:3000 newborns and a prevalence of about 1:4500 [1]. Clinical phenotype encompasses a wide range of manifestations specially involving the skin, nervous system, skeleton and vessels. Very few genotype–phenotype correlations have been identified; for the remaining cases, a broad phonotypical variability is reported among patients and even within the same family [2]. This variability can be, at least in part, ascribed to the inheritance pattern of mutation in the single causative gene NF1 (ch.17q22.1) encoding for the ubiquitous tumor suppressor protein neurofibromin. However, the existence of additional modifier genes has been hypothesized [3,4]. Neurofibromin controls cell-division cycle and differentiation by inactivation of the proto-oncogene KRAS. KRAS is a GTPase responsible for MAPK pathway upregulation, thus promoting cell proliferation, differentiation and migration by modulating the MEK/ERK and phosphatidylinositol 3-kinase—mammalian target of rapamycin signaling pathways. In NF1 patients, the heterozygous pathogenic gene variant is present in every nucleated cell; in case of loss of heterozygosity or when the second wild-type allele is inactivated by a new intra-genic mutation (according to the Knudson two-hit model), neurofibromin becomes inactive or lacking within the cell with repercussion on the RAS-MAPK pathway. Second-hit mutations in the neurofibromin encoding gene have been frequently demonstrated both in the case of NF1-associated tumors and in non-neoplastic NF1-related manifestations [5]. At present, more than 3000 different pathogenic variants of NF1 gene have been described, mostly represented by loss-of-function mutations (ranging from nonsense to missense mutations, from deletions to insertions, from frame-shifts to translocations) [6]. Moreover, although ubiquitous, NF1 is highly expressed in neural crest (NC) derived tissues where it has a prominent regulatory activity on neural stem cell proliferation and precursor migration, with a specific effect depending on the target cell type, arising from each segment of the neural tube (cephalic, vagal, trunk or sacral) [7] (Table 1).

Therefore, it is easy to understand why neuroectoderm-derived lineage cells are the most involved cells in NF1, and why the main disease-related features affect the central nervous system (CNS), peripheral nervous system (PNS) and skin. In spite of a better comprehension of the impact of neurofibromin deregulation on the RAS-MAPK pathway in tumorigenesis (the major cause of reduced life quality and expectancy), the role of NF1 mutation in non-tumor manifestations and its impact on phenotype are less clear at present. The aim of this paper is to provide a comprehensive review of non-oncological neuroradiological manifestations in NF1, with an overview on their possible clinical implications. The methodology adopted for this investigation follows the PRISMA (Preferred Reporting Items for Systematic Reviews and Meta-Analysis) guidelines (see Figure S1 for qualitative study selection process). The search and selection of studies were performed in consensus by two observers; the secondary literature, clinical trials and case reports or series were also included in the search.

## 2. Brain

NF1 patients are known to be prone to CNS tumor development, and the natural history of optic pathway glioma as well as non-optic tumors has been largely described in the literature [8,9,10,11]. Besides tumor-related manifestations, NF1 is also characterized by a wide spectrum of CNS alterations, with variable impacts on functioning and life quality, which can be observed to varying degrees in up to 70% patients [12,13].

### 2.1. Focal Abnormal Signal Intensities: Neuroradiological Tips and Tricks

**Figure 1:**
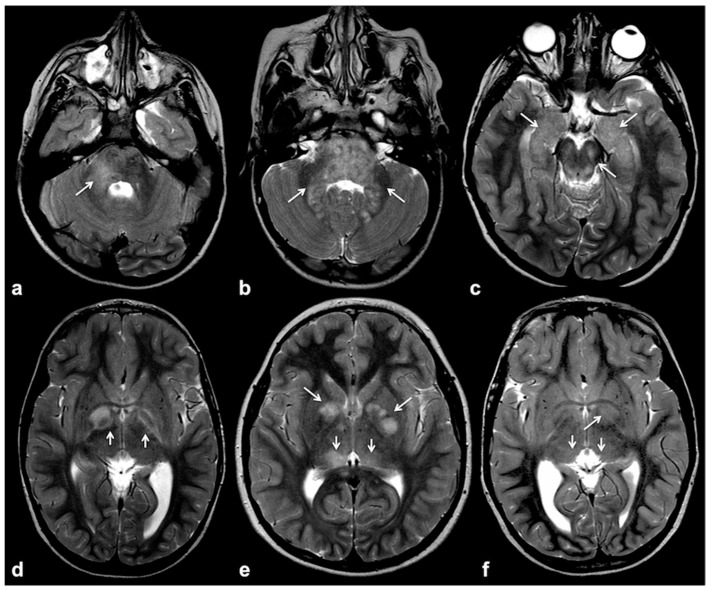
Typical focal abnormal signal intensity (FASI) localization to cerebellar pedicles and cerebellum (**a**,**b**), brainstem, cerebral peduncles and hippocampi (**b**,**c**), as well as basal ganglia (**d**–**f**) on axial TSE T2w MR images (white arrows).

Focal abnormal signal intensities (FASIs), sometimes referred to as unidentified bright objects (UBOs), are focal or diffuse areas of increased T2w signal intensity within brain tissue on magnetic resonance imaging (MRI). Found in about 90% of lifelong NF1 patients, these alterations are most frequently detected in the cerebellum, brainstem and basal ganglia (Figure 1); however, hemispheric and hippocampal lesions may appear over time, suggesting a different pathogenic mechanism compared to other localizations [14,15,16].

FASIs vary in number and size over time, with the highest lesion burden detected in early childhood and the fastest decline observed in the thalami and cerebellum; hemispheric and deep FASIs tend to decrease in number with age, whereas diffuse lesions seem to be more stable over time [17,18,19,20]. Even if the sensitivity of FASI presence in NF1 is high, today they have just been proposed (and not yet accepted) as additional diagnostic criteria due to their relatively low specificity and variable localization; indeed, sensitivity and specificity of FASIs for NF1 averaged 97% and 79%, respectively [21]. In the only literature report of histologic examination, FASIs corresponded to areas of abnormally increased white matter volume corresponding to spongiform myelopathy with myelin vacuolization, but neither marked demyelination nor an inflammatory response [22,23]. These vacuolar changes due to intra-myelinic edema represent the major pathologic finding and could, at least in part, explain their MRI signal. At the MRI examination, FASIs are hyperintense on T2w images and iso-hyperintense on T1w, with no mass effect or post-contrast enhancement. It should be noticed that hyperintensity on T1w is generally limited to FASIs within the basal ganglia, and far less common in the posterior fossa and cerebral white matter, thus suggesting a difference in these localizations [24]; among possible causes of T1-shortening in basal ganglia foci, the most accredited theory is the presence of subtle microcalcification, as documented in previous reports. A possible explanation to this evolution in T1w signal is that calcifications are a late phenomenon due to reparative mechanisms to intra-myelinic edema [22,24].

**Figure 2:**
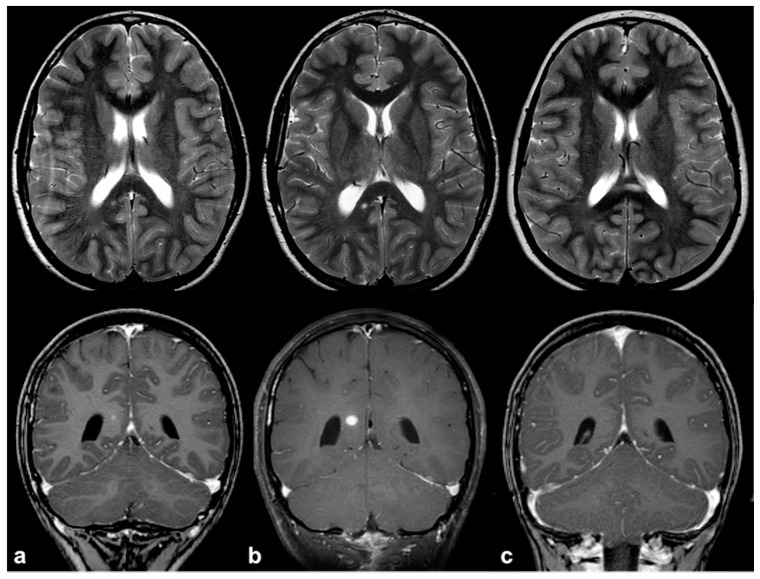
Longitudinal follow-up of atypical brain FASI in a 5 year-old NF1 male patient: axial TSE T2w (first row) and coronal post-contrast T1w (second row) MRI showing a oval-shaped lesion into the right occipital forceps of the splenium corpus callosum at three different time points (**a** = 5, **b** = 5.5, and **c** = 6 years old). The lesion was T2-hyperintense with punctate enhancement at diagnosis, showed a transient volume increase with focal contrast enhancement, and then completely regressed at one-year longitudinal MRI follow-up.

If, in children, FASIs can be easily detected on turbo spin echo (TSE) T2w, in adult patients, fluid attenuation inversion recovery (FLAIR) and proton density (PD) sequences show higher sensitivity; these lesions are generally isointense on T1w and conventional diffusion weighted imaging (DWI), with a sometimes slight elevation in apparent diffusion coefficient values [25]. In rare cases, FASIs can also show atypical MRI features, such as a mild mass effect and focal contrast enhancement (generally transient and regressing over time) (Figure 2); being that these latter features are more typical of brain gliomas, a differential diagnosis with glial tumors can be very challenging.

In this light, advanced techniques can help in the differential diagnosis from glial tumors [15,19,26]. Perfusion-weighted MRI may allow for the detection of tumor-related angiogenesis and increased vascular permeability, features that are usually absent in the case of FASIs and not always present in the case of low-grade glioma. Conversely, spectroscopy has the potential for distinguishing glial tumors (increased choline peak, decreased creatine peak, and absent N-acetylaspartate) from FASIs (where N-acetylaspartate levels are preserved). These spectral abnormalities are also visible in other regions involved in myelin vacuolization, even in absence of areas of patent hyperintensity [27,28]. These findings are further corroborated by microstructural studies, which (in spite of generally silent conventional DWI findings) highlight subtle alterations in fractional anisotropy and radial diffusivity consistent with intramyelin edema; these alterations can persist even after FASI regression [16,18,29,30]. From a prognostic viewpoint, FASIs are benign lesions; in spite of previous evidences suggesting a possible correlation between FASIs’ burden and cognitive impairment, recent studies concluded that they do not represent a strong indicator of executive dysfunction in children with NF1 [20,31]. Indeed, despite conflicting results from different studies [32], to date, FASIs are thought to play a marginal role in microstructural disruption and cognition; conversely, they should rather be considered as a late epiphenomenon of an underlying structural neurodevelopmental disorder [18,29,33]. This consideration may also explain why children with a higher NF1-associated lesion burden seem to have a worse clinical outcome compared to those with a lower lesion burden [20,31,34,35]. Finally, it should be noticed that, despite the fact that clinical management should not be affected by FASIs or optic nerve thickening in asymptomatic patients, their presence frequently results in closer clinical and MRI monitoring (with subsequent unbeneficial effects in terms of cost-effectiveness, as well as emotional burden for families).

### 2.2. Volume, Structural and Functional Connectivity Changes: More Than Meet the Eye

NF1 is characterized by a global increase in brain volume, sometimes associated with macrocephaly, more evident in white rather than in grey matter; these changes seem to be age-dependent and more pronounced in younger patients [23,36,37,38,39]. A significant volume increase is observed in midline structures, with particular reference to the corpus callosum which looks altered both in terms of micro- and macro-structural measurements [37,40]. Subcortical structures, such as the hippocampus, amygdala and basal ganglia (i.e., thalamus and striatum), can also present larger volumes than normal [23,41,42,43,44]. Conversely, grey matter density is lower in the frontal parietal and temporal lobes (and, to a lesser extent, in cingulate and insular regions) with simplified cortical gyration and abnormal cortical thickness that decreases with age [18,23,45,46]. From a micro-structural point of view, these volumetric changes correspond to an extensive global and local white matter disruption, as documented with diffusion tensor imaging (DTI) and a diffusion parameters analysis; indeed, several studies have documented an increased radial diffusivity with reduced fractional anisotropy in lobar white matter, suggesting a link between an impaired microstructure and abnormal fluid accumulation, potentially due to subtle myelin vacuolization [47,48,49]. Focal alterations are more evident in the frontal lobe white matter, [48] and in the anterior thalamic radiation, a white matter bundle connecting the thalamus with frontal lobes that are associated with higher executive functioning [33]. It should be noted that white matter disruption is observed independently from FASIs and other macroscopic NF1-associated lesions, supporting the thesis that axonal degeneration might occur even before, or in the absence of, primary myelination changes [49].

Several functional MRI (fMRI) studies have explored the functional equivalent of the above-described white matter disruption, with particular regard to the evaluation of the more compromised functions in NF1 (namely, executive and visuo-spatial). Both static functional connectivity and resting-state fMRI dynamic properties seemed to be affected in NF1 patients. Alterations in executive functioning correspond to a dysfunction in connectivity through the frontal, superior temporal, parietal and anterior cingulate cortex, with an extensive involvement of the motor, pre-motor and supplementary motor cortex [46,50,51,52]; similarly, for visuo-spatial functioning, a deficient activation of the low-level visual cortex due to anomalous and persistent activation of the default mode network during visual stimulation was observed [53]. Along with these static alterations, the dynamic properties of whole-brain connectivity at the resting-state fMRI were also reduced in NF1 patients compared to healthy controls. These findings are consistent with an overall reduction in the inhibitor neurotransmitter gamma-aminobutyric acid (GABA) levels, both in the occipital and frontal lobes, as documented by few MRI spectroscopy studies [54,55].

The clinical counterparty of the described widespread alterations is represented by the cognitive and behavioral deficits, whose severity may be poorly predicted by resorting to neuroimaging, despite the always more remarkable contribution of advanced MRI techniques. Coordination disorders with reduced visuo-spatial/motor abilities, comprehension deficit, linguistic impairment, autistic mannerisms and attention deficit/hyperactivity disorder represent the most common cognitive manifestations of NF1 [40,41,42,56]. All these findings taken together could probably reflect a delayed or aberrant dendritic pruning depending, at least in part, on NF1 gene mutations; at present, the correlation between impaired function and the involved brain regions is still poorly understood, although these preliminary findings suggest a possible role of these in vivo biomarkers for future disease monitoring and treatment response assessment [18]. However, the variable prevalence and penetrance of these cognitive dysfunctions in NF1 patients plead in favor of the existence of multiple physiopathological mechanisms, which cannot be fully elucidated by morphological and structural neuroimaging approaches alone [44].

### 2.3. Epileptogenic Lesions in NF1

NF1 is associated with higher seizure frequency compared to the general population, with a prevalence of about 5% (lifelong) and increasing incidence with age; clinical manifestations are usually represented by focal seizures onset with secondary generalization [57]. The majority of NF1 patients with seizures presented with focal epileptogenic lesions, and frequently with NF1-related tumors (about 60–65% of cases) [58,59]; however, mesial temporal sclerosis, malformations of cortical development or cerebrovascular lesions have also been sporadically reported as epileptogenic triggers accounting for about 20–25% of all cases [58,59,60], while no clear association is reported with FASI [58,60,61]. In the remaining cases, no focal brain lesion have been documented, and the exact cause of seizure onset in this NF1 patients subgroup is not yet clear [59]. The most reliable hypotheses suggest, as possible mechanisms, an excitation/inhibition imbalance in GABAergic signaling or an abnormal sensitization of ion channels mediated by the RAS-MAPK pathway [62,63,64]. In NF1, epilepsy is generally responsive to medical treatment, whereas in selected cases of medication-refractory, surgery may be envisaged to limit seizure recurrence; in these patients, pre-operative multimodal evaluation (combining electroencephalography, advanced/conventional MRI techniques, and nuclear medicine imaging) can be necessary to distinguish real epileptic trigger from non-epileptogenic lesions [60,65].

### 2.4. Altered Cerebrospinal Fluid Dynamics: Obstructive Hydrocephalus Other Than Tumor-Related

**Figure 3:**
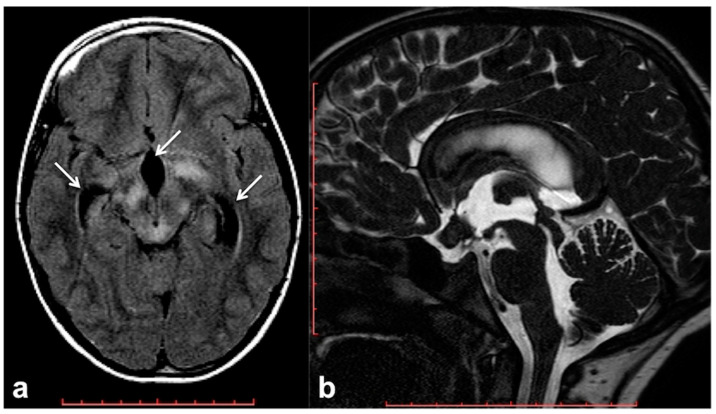
Brain MRI of a 6 year-old boy with NF1 and aqueductal stenosis: axial fluid attenuation inversion recovery (FLAIR) (**a**) showing bilateral basal ganglia and brainstem hyperintensities consistent with FASI. Supratentorial hydrocephalus with enlarged third ventricle and temporal horns of the lateral ventricles was also seen (white arrows); no transependymal CSF flow in periventricular areas was visible, and the fourth ventricle was normal. Sagittal 3D heavily T2-weighted sequence (**b**) revealed the presence of a thickened web in the cerebral aqueduct causing stenosis and hydrocephalus.

Hydrocephalus prevalence in NF1 ranges between 1% in adults and 13% in children, and it is almost invariably represented by non-communicating forms due to the impaired flow of CSF into the ventricular system [66]. Although frequently related to midbrain, diencephalon or basal ganglia masses, non-communicating hydrocephalus can be also due to non-neoplastic lesions. Among these causes of CSF flow alteration, aqueductal stenosis, aqueductal web and superior medullary velum synechiae, whose etiology still remains uncertain, have an increased incidence rate in patients with NF1 compared to the general population [67,68,69]. Symptoms vary depending on pathogenic cause and hydrocephalus entity, despite the fact that asymptomatic incidental dilatation of the ventricles can also be observed [66]. Once ruled out at the imaging of the presence of underlying neoplastic lesions, non-communicating hydrocephalus is generally treated by external shunting or endoscopic third-ventriculostomy (ETV). In the case of triventricular hydrocephalus, ETV represents the golden standard procedure with a high success rate and low risk of post-surgical complications; in some cases, the use of a trans-stoma stent may be required to ensure long-term stoma patency [66,70]. In this setting MRI plays a crucial role both at diagnosis and during the follow-up; besides conventional MRI, the use of 3D heavily T2-weighted sequences provides specific morphologic data regarding CSF pathways, thanks to high spatial resolution (Figure 3), whereas the application of phase-contrast techniques ensures the visualization of CSF dynamics through different compartments (also documenting the post-surgical patency of stoma/stent over time).

### 2.5. CNS Vascular Manifestations of NF1

**Figure 4:**
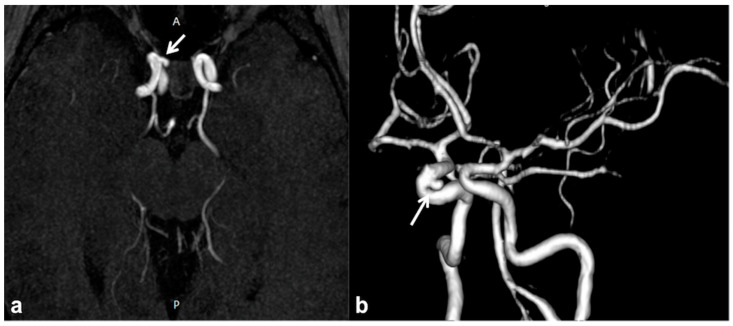
Non-contrast time-of-flight 3D MR angiography maximum intensity projection on the axial plane (**a**) and its volume rendering reconstruction (**b**) in a 10 year-old boy with NF1, showing the presence of a small saccular aneurysm of the right cavernous internal carotid artery (white arrows).

CNS vascular abnormalities are an occasional finding in NF1 patients, with higher prevalence compared to the general population, ranging between 3% and 7% [71]. Cerebral arteriopathy mainly affects the arterial brain supply and, more specifically, the anterior circulation system. This is due to the embryologic origin of the internal carotid artery and its intracranial branches from the NC, whereas the posterior circulation ontogenetically arises from the paraxial mesoderm. Among possible cerebral vasculopathies, moyamoya syndrome (MMS) represents the most common finding, followed by cerebral vascular malformations and aneurysms [72,73,74,75] (Figure 4); all the vascular alterations are not strictly associated with other features of NF1 and do not correlate with disease severity [71].

**Figure 5:**
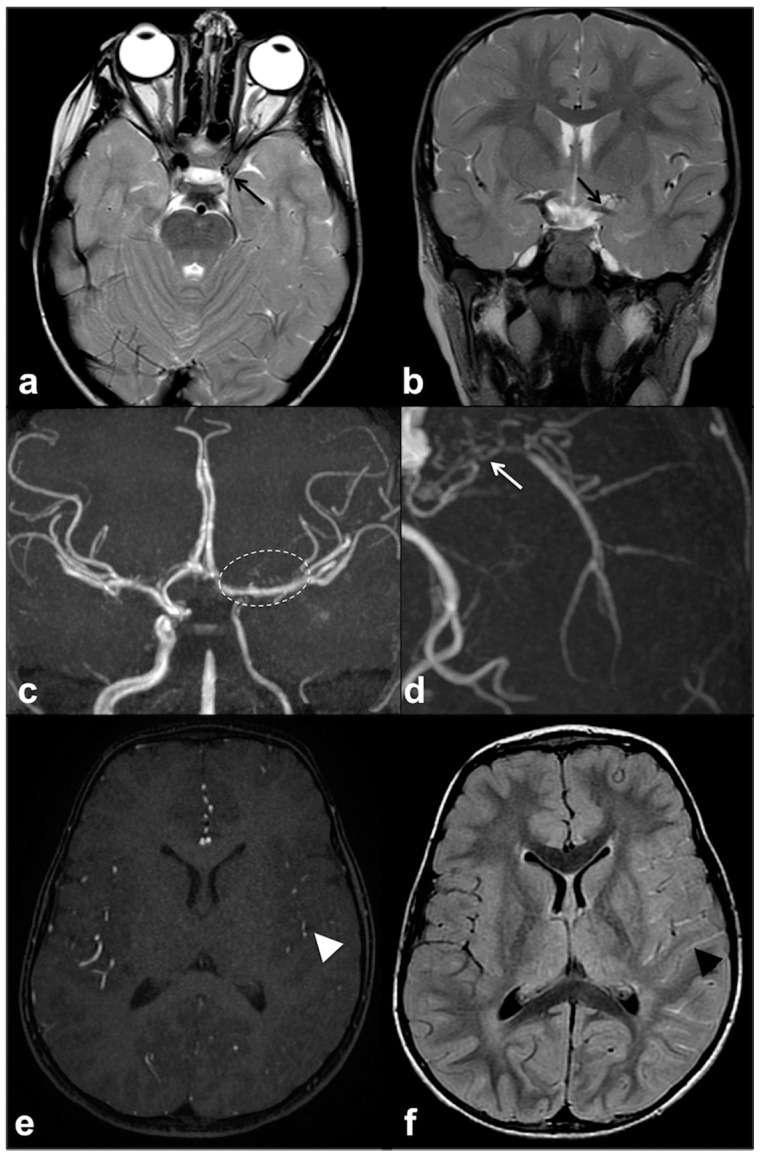
Axial (**a**) and coronal (**b**) TSE T2w MRI of an 8 year-old girl with NF1, showing reduced visualization of left cavernous-supraclinoid internal carotid and proximal left middle cerebral arteries’ flow void (black arrows). Time-of-flight 3D MR angiography maximum intensity projection (**c**) confirmed the severe stenosis of left internal carotid artery and proximal segment of left middle cerebral artery, whereas distal segments are partly supplied by collaterals; dotted line region magnification (**d**) showed multiple small collateral vessels in proximal left middle cerebral artery region (white arrow). Axial time-of-flight 3D MR angiography (**e**) confirmed a reduction in distal middle cerebral artery branches flow (white arrowhead), with a diffuse net of prominent leptomeningeal collaterals with high signal on FLAIR (**f**) due to slow flow (black arrowhead).

MMS is defined as a progressive cerebrovascular angiopathy in the setting of a predisposing condition, such as phakomatoses, caused by narrowing or stenosis in Willis’ polygon arteries, and manifesting as recurrent minor or major strokes. Symptoms are usually represented by acute-onset focal neurological deficits with sudden weakness or numbness in the face, arm or leg on one side; other possible manifestations also include headaches, visual disturbances, developmental delay and seizures [76]. Among phakomatoses, NF1 has been suggested as a possible MMS predisposing disorder, and few susceptibility genetic loci have been recently identified [43,77]. This is why, despite the fact that MMS was usually described as a consequence of cranial radiation therapy for NF1-related optic pathway glioma, the majority of cases have been reported as a primary manifestation of NF1 even in the absence of previous radiotherapy [71,78]. Despite their relatively high prevalence, these manifestations are frequently recognized as poorly symptomatic although potentially fatal; therefore, it may be worth using transcranial Doppler as a screening method for identifying cerebral vasculopathy in children with NF1 and always including unenhanced MR angiography in routine examination protocols of these patients (Figure 5) [79,80].

When clinical and radiological signs of MMS are detected, contrast-enhanced MRI perfusion techniques may represent a useful tool for grading hemodynamic insufficiency and determining the need for surgical revascularization [81]. Investigating vascular abnormalities by the above-mentioned non-invasive methods can help in preventing cerebrovascular complications and selecting patients for subsequent confirmation by more invasive techniques, such as digital subtraction angiography.

## 3. Skull and Orbit

Neuroectodermal cells originating from the cephalic segment of the NC contribute to the development of the craniofacial bone, cartilage and connective tissue, thus participating in the membranous ossification of the skull vault [82]. Being that NF1 is ubiquitously present in these nucleated cells, neurofibromin loss of function leads to defects both in osteo- and chondro-progenitors [82]. Indeed, neurofibromin is highly expressed in growth plate chondrocytes, trabecular osteoblasts, as well as in cells of the periosteum and perichondrium [83], where it plays a crucial role in fibroblast growth factor (FGF) signaling and osteoblast differentiation/mineralization. When mutated, the reduction in neurofibromin expression and/or function results in lower proliferation rates and bone differentiation defect. As previously observed in animal models, the recent histological analysis of bone samples from NF1 patients confirmed a diffuse alteration of the bony architecture caused by a marked reduction in trabecular components [82,83]. The phenotypic equivalent of this pervasive alteration is mainly represented by calvarian defects, bone and dural dysplasia, and orbital manifestation, frequently associated with focal neurological deficits.

### 3.1. Sutural Defects

From a clinical-radiological point of view, sutural defects are generally irregular and sharply demarcated with no sclerosis on the margins; as per other osseous lesions in NF1 patients, the alteration may range in severity and can show progression over time. These abnormalities are most frequently observed in the posterior rather than in the anterior skull, specifically at the lamboid suture [84]. According to the expression of NF1 in NC-derived tissue, it was postulated that calvarial lesions and cranial vault sutures defects were caused by an intrinsic bone development abnormality due to mutations within the gene itself. Some other authors also suggested a possible relation between skull bone defects and increased exogenous pressure due to underlying neurofibromas, progressively leading to bony erosion and sutures’ patency. However, as these benign calvarial lesions were observed both with and without underlying neurofibromas, it remains unclear whether these defects may present a primary or secondary manifestation of the disease [79].

### 3.2. Skull Bone Defects and Associated Dural Dysplasia

**Figure 6:**
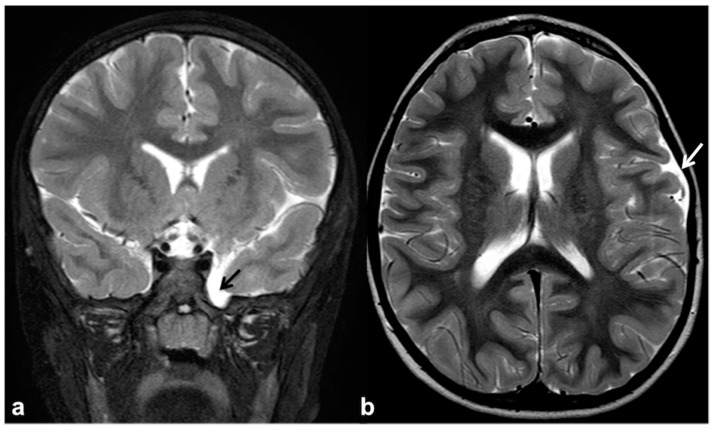
Coronal TSE T2w MRI (**a**) of a 14 year-old girl with NF1, showing left lateral sphenoid meningocele with no associated brain herniation (black arrow). Axial TSE T2w (**b**) of the same patient also showed a smaller dural ectasia of the cranial vault, close to the left temporo-parietal suture (white arrow).

As for sutural defects, the NF1 gene was also proposed to play a role in skull base skeletal development [85]. A large number of craniofacial abnormalities have been reported in NF1. These alterations (more evident in adult patients) range from the most common sphenoid wing dysplasia to rarer findings, such as pure orbital, temporo-mandibular joint (TMJ) and/or dental deformities, a shorter mandible and/or maxilla, and a reduced cranial base length [86,87]. Some authors also suggested a relationship between these skeletal deformities and the presence of dural ectasia [88]. Dural ectasia, defined as a focal dilation of the pachymeningeal layer, is a frequent manifestation of NF1 that can be observed at any point of the dura (Figure 6).

The dural outer layer is strictly connected to the inner periosteal surface of the skull, whereas its inner layer is closely attached to the arachnoid mater; therefore, when bone dysplasia is present, meningocele (and more rarely meningoencephaloceles) can occur in the site of the cranial base defect. These manifestations can progress over time and symptomatically evolve during the course of the disease; the explication of these phenomena can be traced back to alterations in the local cerebrospinal fluid dynamic with the subsequent rising in the intracranial pressure and local mass effect [67].

#### 3.2.1. Sphenoid Wing Dysplasia

**Figure 7:**
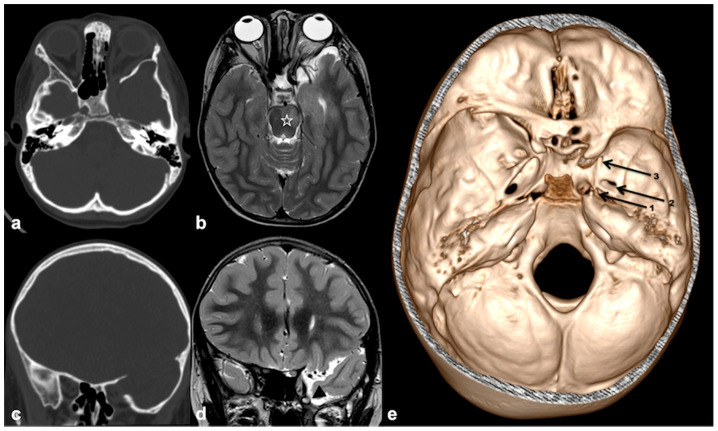
Axial CT (**a**) and TSE T2w MRI (**b**), and coronal CT (**c**) and TSE T2w MRI (**d**) of a 6 years-old boy with NF1, showing left sphenoid wing dysplasia with enlargement of the left orbit (bare orbit sign), bony defect of the posterior orbital wall and enlargement of middle cranial fossa (with associated meningocele—black arrowhead). Brainstem FASIs are also visible on axial TSE T2w MRI (white star). CT volume rendering (**e**) confirmed middle cranial fossa asymmetry with reduced foramina diameter on the affected side (black arrows: 1-carotid canal; 2-foramen ovale; 3-foramen rotundum).

The most characteristic skull abnormality in NF1 patients (>10% cases) is represented by sphenoid wing dysplasia, defined as asymmetric hypoplasia or aplasia of the sphenoid bone, generally affecting the greater wing, sometimes extending to the adjacent structures such as the temporal or occipital bone. At imaging, the most pictorial sign is represented by the absence of the innominate line on the plain radiograph and CT scan (“bare orbit sign”) due to the absence/extreme hypoplasia of the sphenoid wing; this alteration results in enlargement of orbital fissures (conversely the optic canal is generally spared) and elevation of the lesser sphenoid wing. These changes imply widening of the middle cranial fossa and flattening of the posterior aspect of the orbit, with possible herniation of meninges and CSF spaces [89] (Figure 7).

Because of the unilateral sphenoid involvement, the most visible clinical sign is facial asymmetry particularly affecting the orbit. Due to the evolving course of the disease, sphenoid dysplasia and thickening can progress over time causing exophthalmos, incomplete lid closure, corneal ulcerations, and, in the most disabling cases, vision loss. Therefore, periodic monitoring of clinical symptoms and annual radiological assessment of dysplasia progression are crucial for a timely multidisciplinary surgical intervention. This assumption is all the more true given that the association of plexiform neurofibroma to sphenoid dysplasia can occur in more than half of all patients; in these cases, a plexiform tumor is thought to disrupt bone remodeling through complex mechanisms of vascular stealing and osteoclastic paracrine signaling [89]. When extreme sphenoid hypoplasia is present, the occurrence of meningoceles (or more rarely, meningoencephaloceles) at the skull base can be observed. Possible localizations include orbital fissures, posterior skull base sutures, as well as jugular, rotundum and sphenopalatine foramina; Meckel’s cave and inner auditory canal ectasia can also occasionally be observed. In these cases, an annual follow-up is strictly recommended to monitor outpouching enlargement with the subsequent onset of mass effect signs and symptoms [90,91,92].

#### 3.2.2. Imaging Manifestation of Orbital Involvement

NF1-associated orbital manifestations result from a variable combination of three main components: the presence of optic nerve glioma, sphenoid wing dysplasia and peri-orbital plexiform neurofibromas. The posterior aspect of the orbit is generally more affected, making orbit dysplasia a side expression of the underlying sphenoid dysplasia; in rare cases, the other bones forming orbit walls can be primarily affected. Common symptoms are represented by pulsatile exophthalmos (due to the propagation of brain vessels’ systolic pulsations to the eye globe), buphthalmos and visual impairment [86,89]. Ocular proptosis and buphthalmos are typically related to increased intraocular pressure, due both to the presence of sphenoid wing dysplasia (with/without middle cranial fossa herniation) and/or abnormal mesodermal tissue deposition in the canal of Schlemm (the anatomical site of aqueous humor resorption from the anterior ocular chamber) [93]. In this setting, buphthalmos generally underpins the presence of acquired glaucoma; in these cases, neuroimaging (used to rule out the presence of comorbidities such as OPG and neurofibromas) may reveal a posterior impression of the retinal papilla on the ocular bulb (flattening of the fovea and variable optic nerve thinning), clinically corresponding to reduction in visual acuity. Disconjugate eye movements caused by distorted skull development and subsequent strabismus, even in the absence of locally advanced neoplastic lesions, may also contribute to visual disturbance [94].

Due to the age-dependent occurrence of various NF1 related complications, an ophthalmological multidisciplinary assessment every six months is imperative in children until the age of visual maturation, eventually coupled with MRI examinations. After visual maturation, the clinical and radiological assessment can be spaced depending on the patient’s needs in order to ensure an early changes diagnosis, supportive treatments and a prompt surgical approach when needed [95].

#### 3.2.3. Imaging Manifestation of Oral Involvement

In addition to the neurocranium bones, the maxilla and the mandible can sometimes undergo extensive osteolysis and/or hypoplasia; such changes can occasionally also affect the TMJ [89]. Potentially representing the expression of NF1-related developmental dysfunction, these skeletal changes are more commonly observed as secondary pressure atrophy caused by locally advanced neurofibromas arising along the peripheral nerves. These changes may lead to dental abnormalities or later eruption, TMJ dysfunction and skeletal malocclusion, with impairment in the masticatory function triggered by structural factors [90]. In addition, some authors also proposed a link between reduced speech production in NF1 patients with functional disturbances of peripheral nerves and oral overgrowth, although cognitional and learning impairment due to cerebral involvement probably plays a major role in speech aberration [96].

### 3.3. Macrocephaly

Another common finding in NF1 patients is macrocephaly, particularly in infancy where it was proposed as an early feature of the underlying disease. As in other rasopathies, an absolute and relative macrocephaly can be documented in NF1 children and adults. Progressive macrocephaly represents instead a useful clinical indicator for increased intracranial pressure. In this scenario, macrocephaly in NF1 should be considered the epiphenomenon of cerebral growth demodulation and pervasive brain dysfunction related to widespread changes in grey and white matter volumes, specifically affecting midline structures and causing variable neurodevelopmental deficits [42].

## 4. Spine

NF1 can be characterized by a wide spectrum of spinal pathologies. Intra- and paraspinal tumors are the most disabling manifestations, observed in about 40% of patients and usually represented by neurofibromas, leading to variable neurologic dysfunction and spinal deformities [97]. Compared to neurofibromas, intramedullary neoplasms represent only 10% of spinal tumors, the vast majority of which are represented by low-grade gliomas. Here, we describe and report the pathogenesis of NF1-related non-neoplastic manifestations, highlighting the role of imaging-guided diagnostic tools for early identification and disease monitoring.

### 4.1. Medullary UBOs: A Stumbling Block to Neuroimaging

**Figure 8:**
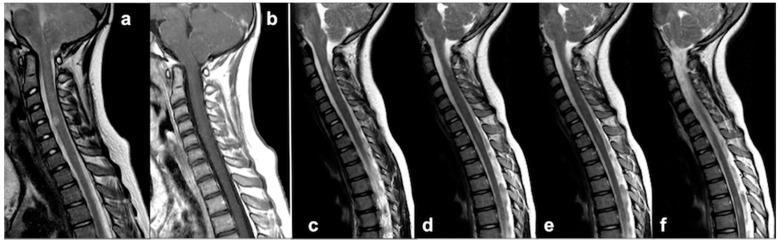
Single and multiple spinal unidentified bright objects (UBOs) in two different NF1 patients. Patient 1: Sagittal TSE T2w (**a**) and contrast-enhanced TSE T1w MRI (**b**) of a 11 year-old boy, showing an isolated and well-defined T2-hyperintense, T1-hypointense, unenhancing lesion localized in the ventral portion of the cervical spine (C4-C5 level). Patient 2: Sagittal TSE T2w at the three most significant levels (**c**–**f**) of a 4 year-old girl, showing multiple spinal lesions disseminated through cervical and thoracic spinal cord.

As previously stated, UBOs in NF1 patients are typically found in the cerebellum (dentate nuclei, cerebellar pedicles and deep lobar white matter) as well as in the brainstem, basal ganglia and thalami; conversely, spinal localization of UBOs has been just recently reported. Indeed, in NF1, intramedullary lesions are generally represented by low-grade tumors, typically astrocytomas (15% patients); at present, only a few reports of sporadic medullary UBOs have been described [98,99]. These single or multiple lesions are described as focal areas of high intensity on T2w and variable signal on T1w MR images, showing neither a significant mass effect nor post-contrast enhancement, almost invariably located into the cervical spinal cord (Figure 8).

Lesions are usually stable at the longitudinal follow up, although in a few cases, spontaneous volumetric reductions were documented, similar to what was described for brain UBOs [98,100]. Despite the lack of histological confirmation, as lesions were never biopsied, being indolent and asymptomatic, authors speculated that these alterations (probably underestimated in previous times) represent focal areas of myelin vacuolization similar to those found in the brain, hence the definition of spinal UBOs [98]. However, due to the limited literature reports, further studies are still required to assess spinal UBOs evolution over time and their possible relationship with the neurological outcome. It would also be useful to analyze the role for specific non-routine MRI sequences in detecting even small UBOs that could be missed at a conventional spine examination [26].

### 4.2. Dural Ectasia, Meningocele and Spinal Deformity: An Etiopathological Continuum

Nearly half of NF1 patients experience severe, lifelong orthopedic manifestations. Among these manifestations, scoliosis and spinal deformities represent the most frequent musculoskeletal finding in NF1, more common in the thoracic region and frequently requiring symptomatic management. Scoliosis and spinal deformities are typically secondary to underlying intra- or extra-spinal abnormalities causing spinal imbalance, such as neurofibromas, bone dysplasia or endocrine disturbances; in a minority of patients, no dystrophic change can be found at the whole-spine examination. Therefore, in these cases, the spinal deformities’ pathogeneses are thought to be similar to idiopathic scoliosis [101]. Among the above-mentioned dystrophic changes, meningocele and dural ectasia probably represent the most pictorial non-neoplastic spinal finding in NF1 patients (with up to 70–80% of dural ectasia and meningoceles found in patients with NF1) [102]. It has been estimated that about 50% of NF1 patients with dural ectasia had a concurrent deformity, most of which are represented by scoliosis [103].

**Figure 9:**
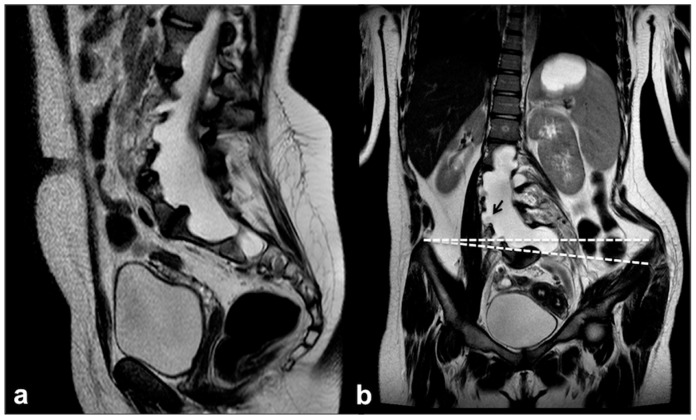
Sagittal (**a**) and coronal (**b**) TSE T2w of a large dural meningocele of the lumbar spine in a 15 year-old girl with NF1 showing: diffuse scalloping of vertebral bodies and pedicles; dural outpunching through the enlarged radicular foramina; periradicular CSF engorgement (black arrows); thoraco-lumbar scoliosis with right lateral pelvis tilt (on the coronal plan—dotted angle).

Dural ectasia and meningocele are different expressions of the same phenomenon, with dural ectasia referring to the circumferential expansion of the dural sac, and meningocele to its localized dilatation. Widening or focal outpouching of the dural sac can herniate through enlarged intervertebral foramina, scalloped vertebral bodies, or defective/absent osseous spinal elements. Similar to spinal neurofibromas (although less extensive), large dural ectasia and meningoceles can also produce a bony erosion of the middle spinal column and posterior neural arch (with the most common feature represented by pedicle deficiency) [104]. At the same time, it should be noticed that in NF1, dural sac dilatation could be also associated with primary bone hypoplasia or aplasia, with alterations falling into the spectrum of spinal dysplasia–dysraphism [105]. When extreme vertebral hypoplasia is present (specifically in the case of bilateral or multiple level involvement), further complications such as spondylolisthesis, non-tumor related spinal canal stenosis and abnormal spinal curvatures could also occur. In these cases, a timely therapeutic approach is strictly required to minimize the risk of permanent deformities, pathologic fractures and pseudo-arthrosis [106]. When dystrophic changes are noted on plain radiographs, being that dural ectasia and meningocele are frequently multiple and not always symptomatic (depending on size and location), MRI represents the golden standard in disease assessment [101] since it allows the optimal visualization of spinal cord, meninges and nerve roots (Figure 9).

In particular, a whole-spine MRI examination was proved to be useful in early identification of occult vertebral dysplasia and in ruling out the presence of intramedullary/paraspinal associated tumors. Nevertheless, in many cases, a CT scan is mandatory for the assessment of spinal bony changes [107]; this is all the more true in case of symptomatic meningocele associated with pervasive bony changes and vertebral instability, which may require multidisciplinary-multistep surgical treatment [108].

The cause of all these manifestations can be traced back to the neural tube defect also implied in the previously mentioned skull vault and base abnormalities, even if the possible contribution of fibroblast dysfunction and aberrant dural pulsation is still debated [7,109]. Indeed, for the spine it is not clear whether vertebral deformities occur before meningeal abnormalities, or conversely whether meningeal ectasia leads to progressive spinal deformities from the early developmental stages [7,109,110]. Therefore, further studies are required to assess whether meningocele should be considered a primary or secondary manifestation of the underlying disorder.

### 4.3. Chiari Malformation and Syrinx

Chiari malformation (CM) refers to a group of complex brain abnormalities affecting the posterior cranial fossa which cause abnormal caudal displacement of the cerebellum and/or brainstem. The two most common variants of CM (notably type I and 1.5) can be occasionally observed in NF1 patients [111,112], with an higher incidence of NF1 among CM patients compared to those observed in the general population (8–11% vs. 0.775%) [111]. The diagnosis of CM is based on MRI evidence of cerebellar tonsils herniation through the foramen magnum (>6 mm) variably combined with brainstem herniation. CM is generally present since birth, although in many cases, it becomes clinically apparent during adulthood, leading to hydrocephalus, crowded small posterior cranial fossa, and, more frequently, spinal syrinx [113]. Several theories have been postulated to explain the etiology of the malformation (i.e., caudal traction, hydrocephalus theory, posterior fossa dysgenesis), with recent evidences strongly arguing in favor of genetic predisposing factors; however, this genetic contribution is still poorly understood and only a few putative genes have been identified at present [114].

Syringomyelia (morphologically defined as a fluid-filled cavity in the central spinal canal at the MRI examination) is frequently observed in symptomatic CM, independent of the CM underlying cause. However, its occurrence in NF1 patients has been observed even in the absence of CM or other possible causes. It remains unsolved whether the syrinx in NF1 should be considered an isolated epiphenomenon or a subtle associated lesion. In this light, because syringomyelia can expand over time, a periodical instrumental follow-up is strongly recommended.

## 5. Conclusions

NF1 is characterized by multisystemic manifestations with the primary involvement of CNS. Although it is the most common tumor-predisposing genetic syndrome, its clinical presentation can be variable and insidious, thus requiring a prompt genetic evaluation. Out of the neuro-oncological complications, on which abundant literature has been produced, there are several pure neuroradiological non-neoplastic findings (both of a malformative and acquired nature) whose pathogenesis, prevalence and natural history still need to be fully elucidated. Some of them probably underlie potentially evolutive disorders that need to be treated as any other clinical manifestations.

## Figures and Tables

**Table 1:** Neural crest (NC) cell populations’ localization, and major derivates during embryologic development.

Location	Neural Crest-Derived Cells
Cranial NC	Chondrocytes, Osteocytes, Odontoblasts (Cranio-facial skeleton) Cranial ganglia, Thyroid cells
Vagal NC	Smooth muscle cells, Cardiac septa, Pericytes (Cardiac development) Enteric ganglia
Trunk NC	Schwann cells Pigmented cells (Melanocytes) Dorsal root ganglia, Sympathetic ganglia Adrenal medulla
Sacral NC	Sympathetic ganglia, Enteric ganglia

## Supplementary Materials

The following are available online at https://www.mdpi.com/article/10.3390/cancers13081831/s1. Figure S1. PRISMA flow diagram of qualitative study selection process (range 1980–at present).

## Abbreviations

CM = Chiari malformation; CNS = central nervous system; CSF = cerebrospinal fluid; DWI = diffusion weighted imaging; ETV = endoscopic third-ventriculostomy; FASI = focal abnormal signal intensity; FGF = fibroblast growth factor; FLAIR = fluid attenuation inversion recovery; fMRI = functional magnetic resonance imaging; GABA = gamma-aminobutyric acid; MMS = moyamoya syndrome; MRI = magnetic resonance imaging; NC = neural crest; NF1 = neurofibromatosis type 1; PD = proton density; PNS = peripheral nervous system; TMJ = temporo-mandibular joint; TSE = turbo spin echo; UBOs = unidentified bright objects.
