# Supplementary material for: Non-Oncological Neuroradiological Manifestations in NF1 and Their Clinical Implications
Source: Cancers (Basel). 2021 Apr 12;13(8):1831. doi: 10.3390/cancers13081831 (PMC8070534; doi:10.3390/cancers13081831)

Supplementary Materials

Figure S1. PRISMA flow diagram of qualitative study selection process (range 1980 – at present).

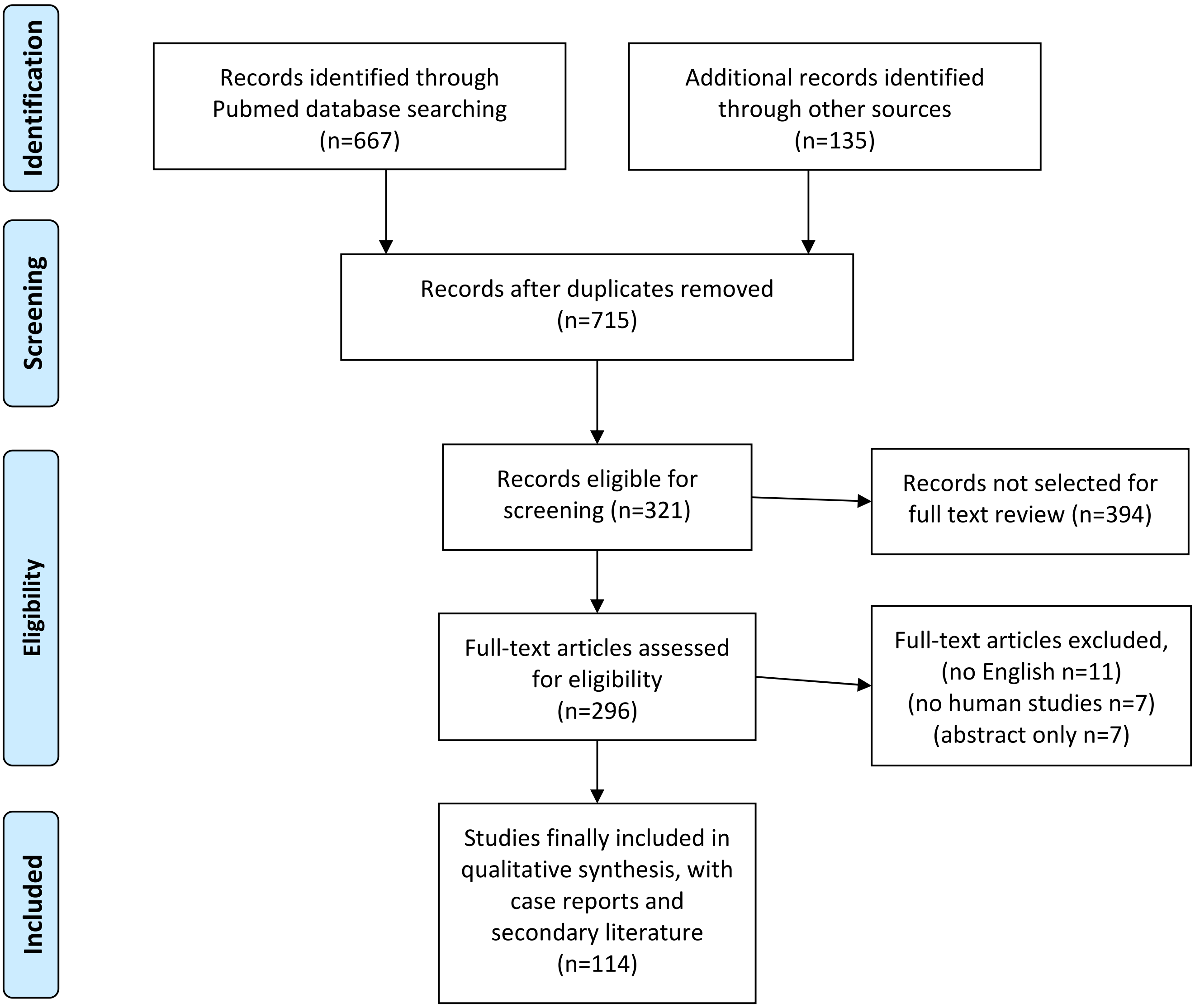

Supplement: Supplementary file 1 [file cancers-13-01831-s001.pdf]
